# The prognostic significance of apical lymph node metastasis in patients with high-risk stage III colon cancer

**DOI:** 10.1038/s41598-022-06054-5

**Published:** 2022-02-08

**Authors:** Kenichi Ishii, Jun Watanabe, Kouki Goto, Yusuke Suwa, Kazuya Nakagawa, Hirokazu Suwa, Mayumi Ozawa, Atsushi Ishibe, Chikara Kunisaki, Itaru Endo

**Affiliations:** 1 Department of Surgery, Gastroenterological Center, Yokohama City University Medical Center, 4-57, Urafune-cho, Minami-ku, Yokohama, 232-0024 Japan; 2 Department of Gastroenterological Surgery, Yokohama City University Graduate School of Medicine, Yokohama, Japan; 3 Department of Surgery, Yokosuka Kyosai Hospital, Yokosuka, Japan

**Keywords:** Cancer, Gastrointestinal cancer, Colorectal cancer

## Abstract

The effect of apical lymph node (APN) metastasis on the prognosis of colon cancer is unknown. The present study investigated the impact of APN metastasis on the prognosis of the patients with high-risk stage III colon cancer. This retrospective multi-institutional study included patients with pathological high-risk stage III colon cancer who underwent surgery between April 2009 and December 2014. Clinicopathological factors were examined by univariate and multivariate analyses to clarify independent risk factors for overall survival (OS) and relapse-free survival (RFS). A total of 185 patients were collected. The 5-year OS rates of patients with and without APN metastasis were 35.0% and 72.1%, respectively (p = 0.0014). The 5-year RFS rates of patients with and without APN metastasis was 16.2% and 57.2%, respectively (p = 0.0002). The rate of distant metastasis in patients with APN metastasis was significantly higher than that in patients without APN metastasis (68.8% vs. 36.7%, p = 0.012). The univariate analysis revealed that the differentiation, lymph node ratio, and APN metastasis were significantly associated with 5-year OS, and the preoperative CEA and CA19-9 levels and APN metastasis were significantly associated with 5-year RFS. The multivariate analysis showed that APN metastasis was an independent risk factor for 5-year OS and RFS. APN metastasis may be independently associated with the prognosis of patients with high-risk Stage III colon cancer.

## Introduction

Lymph node metastasis in colon cancer is an important indicator of the need for postoperative adjuvant chemotherapy^1^ and accurate classification is needed to predict the prognosis of such patients. The American Joint Committee on Cancer (AJCC) staging system and the Union for International Cancer Control (UICC) TNM classification 8th edition recommend the collection of at least 12 lymph nodes for an accurate diagnosis, and lymph node metastasis is categorized completely by number, rather than by location. This classification is simple and easy to understand and has therefore been widely accepted around the world.

However, the Japanese Classification of Colorectal, Appendiceal, and Anal Carcinoma (JCCRC) defines the classification of lymph node metastasis, including information on the number and anatomic location of metastatic lymph nodes; in particular, it places much emphasis on the apical lymph nodes (APNs) around the root of the main feeding blood vessels, and APN metastasis is defined as N3 according to the JCCRC^2^.

In recent years, the importance of APN metastasis for determining the prognosis of colon cancer patients has been debated. The integration of information on APN metastasis into the TMN classification reportedly improves the accuracy of the prognostic prediction of stage III colon cancer patients^3^, and Kang et al. suggested that APN metastasis should be considered a predictive factor for systematic recurrence, especially para-aortic nodal recurrence^4^. According to some reports, APN metastasis is an independent risk factor for the prognosis^5–10^. However, others authors have reported that APN metastasis does not affect the prognosis^11,12^; thus, whether or not APN metastasis influences the overall survival (OS) and relapse-free survival (RFS) rates of patients with colorectal cancer remains unclear. The number of metastatic lymph nodes undoubtedly plays an important role in accurate staging; however, information on the anatomic location of metastatic lymph nodes is also likely an important factor for predicting the prognosis. APN involvement has often been reported to be an independent prognostic factor in colorectal cancer^5–10,13^; thus, the anatomic location of metastatic lymph nodes may complement the UICC staging system^5^.

In addition, the period of adjuvant chemotherapy for stage III colorectal cancer patients has recently been attracting attention. The International Duration Evaluation of Adjuvant Chemotherapy (IDEA collaboration) project suggested that stage III can be divided into T1-3N1 as a low-risk group and T1-3N2 or T4N1-2 as a high-risk group, and although this meta-analysis failed to prove the non-inferiority of 3-month vs. 6-month adjuvant chemotherapy for patients with stage III colon cancer in terms of OS, there was only a 0.4% difference in 5-year OS between the 2 groups^14^. Furthermore, among high-risk stage III patients who received adjuvant chemotherapy with capecitabine and oxaliplatin (CAPOX), there was only 1% difference in the 5-year OS rates of patients who received a 3-month regimen and those who received a 6-month regimen; thus, the report supported the 3-month administration of adjuvant CAPOX for most stage III colon cancer patients. However, whether or not three months of CAPOX therapy is adequate for patients with APN metastasis, whose prognosis has been said to be especially poor, is unclear.

Differences in patient background characteristics among reports may be the reason for the lack of clarity regarding whether or not APN metastasis affects the prognosis of colon cancer patients. We therefore focused on high-risk stage III patients in the present study. The present study investigated the influence of APN metastasis on the prognosis of patients with high-risk stage III colon cancer.

## Methods

This was a multicenter, retrospective study of patients with colon cancer who underwent surgery at our three institutions from April 2009 to December 2014. This study protocol was approved by the Ethics Advisory Committee of Yokohama City University Medical Center and the institutional review board of each participating hospital before the study was initiated. Yokohama City University Medical Center, Yokohama City University Hospital, and Yokosuka Kyosai Hospital participated in this study.

Patient data were collected from clinical reports from each institution. In order to align the patient backgrounds, the inclusion criteria were T1-3N2 or T4N1-2 in high-risk stage III patients diagnosed based on operative findings and the examination of the resected specimen. The objects were primary cancer of the cecum and ascending, transverse, descending, and sigmoid colon or rectosigmoid region, similar to the adjuvant chemotherapy for colon cancer with high evidence (ACHIEVE) trial, which is an IDEA project^15^. The exclusion criteria were multiple malignant tumors without surgical resection, recurrent cases, cases that did not undergo curative resection, and cases with distant metastasis or emergent surgery. There were 603 patients who underwent radical operations and who were diagnosed with stage III colorectal cancer according to the TNM classification (UICC) based on a postoperative histopathological examination, and there were 185 applicable patients diagnosed with high-risk stage III disease (T4, N2, or both). These patients were classified according to the absence or presence of APN metastasis and their background characteristics and prognostic factors were examined.

### Surgery and adjuvant chemotherapy

All patients underwent D3 dissection, including dissection of the APNs. The right-sided colon was defined as the part from the cecum to the splenic flexure of the transverse colon, and the boundary of the APNs was defined as the left margin of the superior mesenteric vein. The left-sided colon was defined as the part from the descending colon to the rectosigmoid colon, and the lymph nodes around the root of the inferior mesenteric artery on the ventral side were excised and defined as APNs. Postoperative chemotherapy based on 5-fluorouracil was considered for all stage III patients. The attending doctors decided whether or not to perform postoperative chemotherapy based on the general condition and compliance of colon cancer patients.

### Pathologic examinations

The regional lymph nodes were individually removed from the specimen immediately after surgery manually by the surgeons and were classified as pericolic lymph nodes, intermediate lymph nodes, or APNs. They were pathologically evaluated by the pathologists in the gastrointestinal tract according to the UICC diagnostic criteria. The lymph node ratio was defined as the number of metastatic lymph nodes divided by the total number of dissected lymph nodes.

### Follow-up

Postoperative surveillance methods included collection of the medical history, physical examinations, laboratory studies (including serum carcinoembryonic antigen[CEA] and carbohydrate antigen 19-9[CA19-9] every three months), and CT examinations of the chest to abdomen and pelvis every six months for the first three years; follow-up was then performed every six months for the next two years. Colonoscopy was performed in the first, third, and fifth year after the operation. The diagnosis of recurrence was made using clinical, radiographic, and histological methods. Local recurrence was defined as tumor recurrence around the anastomotic site or the region of the primary operation. Distant metastasis was defined as metastasis to the distant organs with hematogenous or lymphogenous spreading, diffuse peritoneal seeding, or metastasis in all other nonregional lymph nodes, such as the paraaortic lymph nodes.

### Statistical analyses

Categorical variables are reported as numbers and percentages, and continuous variables are reported as medians and interquartile ranges (IQRs). The following continuous variables were divided into 2 categories for the analyses: age (≥ 65 years, < 65 years), BMI (≥ 25 kg/m^2^, < 25 kg/m^2^), preoperative CEA level (≥ 5 ng/ml, < 5 ng/ml), preoperative CA19-9 level (≥ 37 ng/ml, < 37 ng/ml), tumor size (≥ 50 mm, < 50 mm), and lymph node ratio (≥ 0.15, < 0.15). Five-year OS was defined as the time from the first treatment to death for any reason within 5 years, or the last follow-up examination. RFS was defined as the time from the initial treatment to the date on which recurrence or distant metastasis was diagnosed, or the last follow-up examination.

Statistical analyses were performed using the JMP Pro software program (version 15.0.0, SAS Institute Inc., Cary, NC, USA). The differences in variables for each category were analyzed by Pearson’s chi-squared test, Fisher’s test, or Wilcoxon’s rank sum test, and the 5-year OS and RFS rates were shown using the Kaplan–Meier method, with the log-rank test used to compare prognostic factors and survival curves. A Cox regression analysis was used to determine the hazard ratio. *P* values of < 0.05 were considered to indicate statistical significance. A Cox proportional hazards model was used to identify independent prognostic risk factors for 5-year OS and RFS.

## Results

Table 1 shows the clinicopathological data of the patients with high-risk stage III colon cancer. The median age was 71 (IQR: 61–78) years, 63.2% (117/185) of the patients were male and 36.8% (68/185) of the patients were female. The median BMI was 22 (19.9–24.3). The median preoperative serum CEA level was 5.0 (IQR: 2.6–11.1) ng/mL, and the median CA19-9 level was 12 (IQR: 6–26.5) ng/mL. The median maximum tumor size was 45 (IQR: 35–60) mm. Right-sided colon cancer was seen in 38.4% (71/185) of the patients and left-sided colon cancer was seen in 61.6% (114/185) of the patients. A laparoscopic approach was used for treatment in 55.1% (102/185) of the cases. On histological examination, 27 tumors were poor or mucinous, and 158 tumors were well-differentiated or moderately differentiated adenocarcinoma. The median number of retrieved lymph nodes was 25 (IQR: 18–33), and the median number of metastatic lymph nodes was 4 (IQR: 1–5). Postoperative adjuvant chemotherapy was administered to 62.16% (115/185) of the patients.

**Table 1:** Correlation between the APN metastasis and the clinicopathologic factors.

Variable	APN positive n = 16	APN negative n = 169	Total n = 185	P value
Age (years) ^a^	72 (58–78)	71 (61–78)	71 (61–78)	0.717
Male sex	13 (81.3)	104 (65.5)	117(63.2)	0.118
BMI^a^	20.7(19.5–23.7)	22.3(19.9–24.3)	22 (19.9–24.3)	0.318
**PS**				0.500
0	15 (93.8)	150 (88.7)	165 (89.2)	
1	1 (6.2)	16 (9.5)	17 (9.2)	
2	0 (0)	1 (0.6)	1 (0.5)	
3	0 (0)	2 (1.2)	2 (1.1)	
CEA^a^	4.2 (2.7–9.3)	4.9 (2.5–11.2)	5.0 (2.6–11.1)	0.401
CA19-9 ^a^	12 (6.0–52)	12 (6.2–26)	12 (6.0–26.5)	0.848
Tumor size^a^, mm	57.5 (30–83)	45 (35–60)	45 (35–60)	0.237
**Location**				0.187
Cecum	0 (0)	18 (100)	18 (9.7)	
Ascending colom	4 (25.0)	24 (14.2)	28 (15.1)	
Transverse colon	4 (25.0)	21 (12.4)	25 (13.5)	
Descending colon	0 (0)	9 (5.3)	9 (4.9)	
Sigmoid colon	3 (18.8)	62 (36.7)	65 (35.1)	
Rectosigmoid	5 (31.3)	35 (20.7)	40 (21.6)	
Right-sided colon	8 (50.0)	63 (37.3)	71 (38.4)	0.317
Left-sided colon	8 (50.0)	106 (62.7)	114 (61.6)	
**Differentiation**				0.993
Well	4 (25.0)	44 (26.0)	48 (25.9)	
Moderate	10 (62.5)	100 (59.2)	110 (59.5)	
poor	1 (6.3)	12 (7.1)	13 (7.0)	
mucinous	1 (6.3)	13 (7.7)	14 (7.6)	
**pT category**				0.552
T1	0 (0)	2 (1.2)	2 (1.1)	
T2	1 (6.3)	4 (2.4)	5 (2.7)	
T3	5 (31.3)	35 (20.7)	40 (21.6)	
T4	10 (62.5)	128 (75.7)	138 (74.6)	
**pN category**				0.0139
N1	3 (18.8)	87 (51.5)	90 (48.6)	
N2	13 (81.3)	82 (48.5)	95 (51.35)	
Harvested LNs ^a^	27 (22.5–33.5)	24 (18–32)	25 (18–33)	0.683
Metastatic LNs ^a^	8 (5.8–11.8)	3 (1–5)	4 (1–5)	< 0.0001
LN ratio ^a^	0.3 (0.2–0.4)	0.14(0.06–0.22)	0.14(0.07–0.25)	< 0.0001
Ly +	8 (50.0)	128 (75.7)	136 (73.5)	0.026
V +	13 (81.3)	123 (72.8)	136 (73.5)	0.463
**Type of surgery**				0.666
Laparoscopic	8 (50.0)	94 (55.6)	102 (55.1)	
Open	8 (50.0)	75 (44.4)	83 (44.9)	
Operation time(min)	199.5 (183–223)	181 (148–214)	183 (149–216)	0.112
Bleeding(ml)	45 (9–426)	47.5 (10–150)	47.5(10–161)	0.580
Complication ^b^	4 (25)	41 (24.3)	45 (24.3)	0.948
Adjuvant chemotherapy	9 (56.3)	106 (62.7)	115 (62.2)	0.610
Recurrence	12 (75.0)	69 (40.8)	81 (43.8)	0.009
Local	1 (6.3)	7 (4.1)	8 (4.3)	0.692
Distant sites	11 (68.8)	62 (36.7)	73 (39.5)	0.012
Liver	5 (31.3)	26 (15.4)	31 (16.8)	0.104
Lung	3 (18.8)	11 (6.5)	14 (7.6)	0.077
Peritoneal	2 (12.5)	18 (10.7)	20 (10.8)	0.820
Other sites	2 (12.5)	8 (4.7)	10 (5.4)	0.189

Values in parentheses are percentages, unless indicated otherwise; ^a^Values are median (IQR: first quartile—third quartile).

BMI: body mass index, calculated as weight in kilograms divided by height in meters squared, PS: performance status, CEA: carcinoembryonic antigen, CA19-9: carbohydrate antigen 19-9, LN: lymph node, LN ratio: the number of metastatic lymph nodes divided by the total number of dissected lymph nodes, Ly+: lymphatic involvement, V+: vascular invasion.

^b^ Clavien-Dindo classification grade ≥ II, within 30 days after surgery.

The patients with high-risk stage III colorectal cancer were divided into two groups according to the presence or absence of APN metastasis and their background characteristics were compared. A total of 8.6% (16/185) of patients were positive for APN metastasis. The number of metastatic lymph nodes in patients with APN metastasis was significantly greater than that in the patients without APN metastasis (8 vs. 3, p < 0.0001), as was the lymph node ratio (0.3 vs. 0.14, p < 0.0001). Consequently, a significant proportion of patients with APN metastasis were diagnosed with pN2. The group with APN metastasis had a significantly higher recurrence rate than the group without (with APN metastasis vs. without APN metastasis: 75.0% vs. 40.8%, p = 0.009), and while there was no significant difference in the rate of local recurrence (6.25% vs. 4.14%, p = 0.692), there was a significant difference in the rate of distant metastasis (68.75% vs. 36.68%, p = 0.012), with no significant difference noted between the two groups in the rates of liver, lung, and local site recurrence. The liver (31.3%) was the most common site of distant metastasis in high-risk stage III patients with APN metastasis, as noted in a previous report^16^.

**Figure 1:**
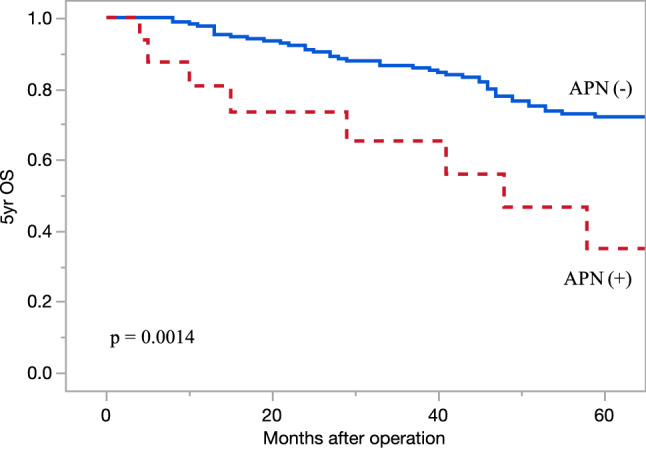
Kaplan–Meier curves showing the 5-year OS rates of high-risk stage III patients with and without APN metastasis. Outcomes of patients with high-risk stage III colorectal cancer. The 5-year OS rates of patients with and without APN metastasis were 35.0% and 72.1%, respectively (p = 0.0014). OS: overall survival, APN: apical lymph node.

**Figure 2:**
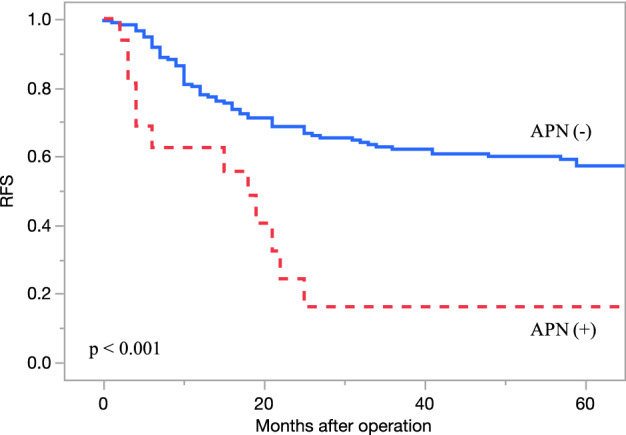
Kaplan–Meier curves showing the 5-year RFS rates of high-risk stage III patients with and without APN metastasis. The outcomes of patients with high-risk stage III colorectal cancer. The RFS rates of patients with and without APN metastasis were 16.2% and 57.2%, respectively (p = 0.0002). RFS: relapse free survival, APN: apical lymph node.

Regarding the operative results, there was no significant difference in the operation time, amount of intraoperative bleeding, open or laparoscopic approaches, or complications. The patients were followed up for a median 58 (IQR: 37–69) months, and the 5-year OS and RFS rates were 69.4% and 54.0%, respectively. The OS differed significantly between the groups; the 5-year OS rates of patients with and without APN metastasis were 35.0% and 72.1%, respectively (p = 0.0014, Fig. 1). The RFS also differed significantly between the groups; the RFS of the patients with and without APN metastasis was 16.2% and 57.2%, respectively (p = 0.0002, Fig. 2).

The distribution of potential prognostic factors according to the absence or presence of APN metastasis is displayed in Table 2. The univariate analysis indicated that the factors associated with the 5-year OS of the patients were differentiation, lymph node ratio, and APN metastasis. The factors associated with 5-year RFS were the preoperative CEA, CA19-9 level, and APN metastasis. In the univariate analysis, the common risk factor for 5-year OS and RFS was APN metastasis (Table 3).

**Table 2:** Univariate analyses of risk factors for 5-year OS and RFS.

Variables	5-year OS			RFS		
	HR	95%CI	*p* value	HR	95%CI	*p* value
Age, ≥ 65 years	1.3	0.72–2.4	0.37	0.85	0.54–1.3	0.47
Sex, Male	1.3	0.88–1.8	0.21	1.2	0.78–1.9	0.37
BMI ≥ 25	1.2	0.64–2.4	0.54	1.0	0.59–1.8	0.93
PS, ≥ 1	1.1	0.47–2.6	0.81	0.68	0.29–1.6	0.36
CEA, ≥ 5 ng/dl	1.6	0.89–2.7	0.12	1.8	1.1–2.8	0.011
CA19-9, ≥ 37 ng/dl	1.8	0.96–3.3	0.07	1.7	1.0–2.8	0.047
Tumor size, ≥ 50 mm	1.2	0.71–2.1	0.46	1.3	0.82–2.0	0.28
Location, Right-side	1.2	0.68–2.1	0.53	1.1	0.67–1.7	0.82
Differentiation, poorly or mucinous	2.8	1.5–5.2	0.002	1.4	0.77–2.5	0.28
pT, T4	1.3	0.66–2.5	0.46	1.5	0.87–2.6	0.15
pN, N2	1.2	0.68–2.0	0.57	1.1	0.72–1.7	0.62
LN ratio, ≥ 0.15	2.0	1.1–3.5	0.019	1.5	0.95–2.3	0.086
lymphatic involvement,	1.1	0.56–2.0	0.88	0.76	0.47–1.2	0.25
vascular invasion	1.4	0.70–2.6	0.37	1.4	0.82–2.3	0.24
APN metastasis	3.2	1.5–6.8	0.003	3.0	1.6–5.6	< 0.001
Adjuvant chemotherapy	0.64	0.37–1.1	0.11	0.69	0.44–1.1	0.10

OS: overall survival, RFS: relapse free survival, HR: hazard ratio, CI: confidence interval, BMI: body mass index, calculated as weight in kilograms divided by height in meters squared, PS: performance status, CEA: carcinoembryonic antigen, CA19-9: carbohydrate antigen 19-9, pT: pathological T stage, pN: pathological N stage, LN: lymph node, LN ratio: the number of metastatic LN divided by that of retrieved LN, APN: apical lymph node.

**Table 3:** Multivariate analyses of risk factors for 5-year OS and RFS.

Variables	5-year OS			RFS		
	HR	95%CI	*p* value	HR	95%CI	*p* value
CEA, ≥ 5 ng/dl				1.7	1.0–2.6	0.035
CA19-9, ≥ 37 ng/dl				1.3	0.77–2.2	0.32
Differentiation, poorly or mucinous	3.0	1.5–5.7	0.0013			
LN ratio, ≥ 0.15	1.5	0.79–2.7	0.22			
APN metastasis	3.3	1.4–7.4	0.0052	2.9	1.6–5.4	0.0008

OS: overall survival, RFS: relapse free survival, HR: hazard ratio, CI: confidence interval, CEA: carcinoembryonic antigen, CA19-9: carbohydrate antigen 19-9, LN ratio: the number of metastatic LN divided by that of retrieved LN, APN: apical lymph node.

A multivariate Cox regression analysis identified the following independent risk factors for poor 5-year OS: APN metastasis (hazard ratio[HR] = 3.3; 95% confidence interval[CI] = 1.4–7.4, p = 0.0052) and histologically poor or mucinous tumor (HR = 3.0, 95% CI = 1.5–5.7, p = 0.0013), and those for short RFS were APN metastasis (HR = 2.9, 95% CI = 1.6–5.4, p = 0.0008) and serum CEA (HR = 1.7, 95% CI = 1.0–2.6, p = 0.035) after adjustment for confounding variables. In the multivariate logistic regression analysis, APN metastasis was statistically the strongest predictor of a poor prognosis in patients with high-risk Stage III colon cancer.

## Discussion

The influence of APN metastasis on the prognosis and recurrence of patients with colon cancer remains controversial. The number of metastatic lymph nodes is one of the most important factors in determining the need for adjuvant chemotherapy for colorectal cancer patients, and it is also an important factor in predicting the prognosis. The AJCC and UICC TMN classification classify the pN stages based on the number of metastatic lymph nodes; this system is easy to understand and is used in most of the world. Suzuki et al. showed that the number of metastatic lymph nodes predicted the prognosis better than the distribution of metastatic lymph nodes in patients with stage III colon cancer^12^, and some authors have shown no marked difference in the survival of patients with stage N2 disease and those with APN metastasis^17,18^.

In contrast, some reports have suggested the influence of the distribution of metastatic lymph nodes on the prognosis. Huh et al. proposed the concept that the metastatic lymph node distribution is an independent prognostic factor for both OS and disease-free survival in patients with sigmoid colon and rectal cancer^6^. Chen et al. showed that the T3 and T4 stages and poor differentiation were significant correlated with APN metastasis in 578 colorectal cancer patients^19^. Reportedly, the greater the number of metastatic lymph nodes in patients with APN metastasis, the poorer their prognosis^13^. In the present analysis, the number of metastatic lymph nodes increased in the presence of APN metastasis (p < 0.0001); thus, the proportion of N2 cases increased. In addition, the 5-year OS and RFS rates of high-risk stage III patients with APN metastasis were significantly worse than those of patients without APN metastasis. The results of the Cox regression analysis in this study showed that APN metastasis is an independent risk factor for RFS, and it is meaningful to distinguish APN metastasis from other lymph node metastases.

APN metastasis reportedly represents a local—rather than systemic—problem^18,20^. In contrast, Kang et al. reported that colorectal cancer patients with APN metastasis had a much higher rate of systemic recurrence rate than those without APN metastasis (48.5% vs. 20.8%, p < 0.001), whereas there the local recurrence rates of the groups did not differ to a statistically significant extent^4^. Huh et al. revealed that the 5-year OS rate of stage IV patients with left-sided colorectal cancer was not significantly different from that of stage III patients with APN metastasis (45% vs. 40%, respectively, p = 0.761)^6^. Our analysis showed that high-risk stage III patients with APN metastasis had a poor prognosis, which was in line with previous reports^3–10^. The 5-year OS rate in high-risk stage III patients with APN metastasis was only 35.0%, and the distant metastasis rate of high-risk stage III patients with APN metastasis was significantly higher than that of patients without APN metastasis (68.8% vs. 36.7%, respectively; p = 0.0012). These results suggest that APN metastasis is an indication of systemic disease rather than a regional problem. Therefore, distinguishing APN metastasis from other lymph node metastasis may benefit patients with high-risk stage III colon cancer, as information concerning APN metastasis will help predict the OS and RFS according to patient risk factors and may help guide the administration of adjuvant chemotherapy.

APN metastasis was diagnosed in 8.6% of our patients, and the rate was similar to that of previous reports; the incidence of lymph node metastasis at the root of the inferior mesenteric artery was reported to be 0.3–13.5%^4,6,11,21^ in patients with left-sided colorectal cancer and 3.0–17.1%^9,10,18^ in patients with right-sided colon cancer. In this analysis, only 6 of 16 patients (37.5%) were diagnosed with APN metastasis by preoperative CT; thus, it may be difficult to diagnose APN metastasis preoperatively. Therefore, it is meaningful to perform high-level ligation and D3 dissection for patients with advanced cancer, even if there is no obvious APN metastasis on preoperative CT.

As another topic, it was reported that elevated postoperative CEA—especially within the first 12 months after surgery—is an important indicator of recurrence of colon cancer^22^. In this study, the blood levels of CEA were followed for 5 years, which helped to indicate when to perform additional tests to detect the recurrence of colorectal cancer. Therefore, it is thought that routine postoperative CEA measurement should be recommended.

According to a large-scale recent study, the HRs for 5-year OS and RFS high-risk stage III patients who received 6-month and 3-month postoperative adjuvant chemotherapy with CAPOX only showed a slight difference, suggesting that 3-month postoperative adjuvant chemotherapy is acceptable^14^. In our institutions, patients diagnosed with stage III disease are considered for adjuvant chemotherapy with 5-fluorouracil based on their performance status, social background, and personal wishes. Even though the adjuvant chemotherapy regimens in our study were not unified and consisted of several regimens (e.g., CAPOX, fluorouracil, leucovorin and oxaliplatin[modified FOLFOX6], S-1 and oxaliplatin[SOX], and uracil and tegafur plus leucovorin[UFT/LV], and despite the fact that the percentage of high-risk stage III patients with APN metastasis who received adjuvant chemotherapy was relatively low (56.3%) in comparison to a previous study (50%-93.9%)^6,18,23^, the high-risk stage III patients with APN metastasis still had a significantly worse prognosis than those without APN metastasis, and a multivariate analysis showed that APN metastasis was independently associated with a worse prognosis. Therefore, APN metastasis should be distinguished from metastasis of other lymph nodes, and it may be best to avoid shortening the postoperative adjuvant chemotherapy regimen, especially for high-risk stage III patients with APN metastasis. Furthermore, it was reported that even though patients with APN metastasis received standard FOLFOX adjuvant chemotherapy, they had poorer disease-free survival and OS in comparison to those without APN metastasis^16^; thus, future studies should explore the optimum adjuvant chemotherapy regimen for patients with APN metastasis.

The present study was associated with some limitations. First, this was a retrospective study, and the number of patients with APN metastasis was very small. Second, the prognosis reportedly differs between right- and left-sided colon cancers^24^; however, we did not consider the location in this analysis because the number of the high-risk stage III patients with APN metastasis was very small, and rectal cancers were excluded. Third, the adjuvant chemotherapy regimens were not identical, and the percentage of patients who received postoperative chemotherapy was relatively low, which may have influenced the results to a degree and hindered an appropriate analysis.

In conclusion, even though the study population was limited to patients with high-risk stage III disease, APN metastasis might be an independent predictor of the prognosis, and not only the number of metastatic lymph nodes but also the location of the metastatic lymph nodes might be deemed important for the prognosis of colon cancer patients. Although the number of patients with APN metastasis is relatively small, their poor prognosis should not be ignored. Further data on APN metastasis may help provide a more detailed prediction of the prognosis of colon cancer patients, in addition to the information obtained through the TNM staging system. Since the prognosis of high-risk stage III patients tends to be worse if they have APN metastasis, it may be better to monitor them closely and not shorten the duration of adjuvant chemotherapy.

### Ethics approval

This study was performed in line with the principles of the Declaration of Helsinki. This study protocol was approved by the Ethical Advisory Committee of Yokohama City University Medical Center and the institutional review board of each participating hospital before the study was initiated. Yokohama City University Medical Center, Yokohama City University Hospital, and Yokosuka Kyosai Hospital participated in this study.

### Consent for participate and publication

Due to the retrospective nature of the study, written informed consent was not obtained. We used the opt-out approach to disclose the study information. We obtained an "informed consent waiver" from the Ethical Advisory Committee of Yokohama City University Medical Center.
